# Fitspiration, Thinspiration, Body Positivity, and Body Neutrality Contents on Image‐Based Social Media: Associations With Body Image, Mood, Self‐Esteem, and Disordered Eating Behavior in Women With and Without Self‐Reported Eating Disorders—An Ecological Momentary Assessment Study

**DOI:** 10.1002/eat.70027

**Published:** 2026-01-08

**Authors:** Kristine Schönhals, Christopher Lalk, Silja Vocks

**Affiliations:** ^1^ Institute of Psychology, Department of Clinical Psychology and Psychotherapy Osnabrück University Osnabrück Germany; ^2^ Institute of Psychology, Department of Psychotherapy Research and Clinical Psychology Osnabrück University Osnabrück Germany

**Keywords:** appearance comparisons, body image, body neutrality, body positivity, eating disorders, ecological momentary assessment, fitspiration, self‐esteem, social media, thinspiration

## Abstract

**Objective:**

This study examined everyday exposure to fitspiration, thinspiration, body positivity, and body neutrality content on image‐based social media and its associations with body image, mood, self‐esteem, and disordered eating behavior using ecological momentary assessment (EMA) in women with and without eating disorders.

**Method:**

Women with self‐reported anorexia nervosa or bulimia nervosa (*n* = 62) and women without eating disorders (*n* = 81) reported their social media use, mood, body image, self‐esteem, appearance comparisons, and disordered eating behavior via a smartphone app for 7 days.

**Results:**

Viewing fitspiration and thinspiration content was significantly associated with lower happiness, higher body dissatisfaction, and lower body appreciation, but not with daily‐reported restrained eating. Only thinspiration content was significantly associated with higher sadness and lower self‐esteem. Upward appearance comparisons mediated the associations of exposure to fitspiration and thinspiration content with all outcomes. Except for higher body appreciation after viewing body neutrality content, there were no main effects of body positivity and body neutrality. Compared to women without eating disorders, those with self‐reported anorexia nervosa or bulimia nervosa showed a greater reduction in self‐esteem after viewing thinspiration.

**Discussion:**

The results highlight the possible detrimental effects of exposure to fitspiration and thinspiration content on mood, body image, and self‐esteem in women with and without self‐reported anorexia nervosa or bulimia nervosa alike, while only body neutrality content was related to higher body appreciation. Women with and without eating disorders should be educated about the possible negative influences of content purporting to improve one's appearance.

## Summary

- This study addresses the associations of popular contents on social media, that is, fitspiration, thinspiration, body positivity, and body neutrality, with mood, body image, self‐esteem, and restrained eating in everyday life.
- The results indicate that thinspiration in particular, but also fitspiration, is related to negative outcomes (e.g., lower happiness and body appreciation, higher body dissatisfaction), while no associations of body positivity or body neutrality with positive outcomes emerged, except for higher body appreciation after viewing body neutrality content.
- Potential negative effects of thinspiration and fitspiration content on social media should be communicated both to women with self‐reported anorexia nervosa or bulimia nervosa and women without eating disorders.

## 1 Introduction

In recent decades, appearance pressures from social media have emerged as a new risk factor for negative body image (Roberts et al. 2022), which is in turn associated with the development of eating disorders (EDs; Stice and Shaw 2002). Specifically, there is growing evidence to suggest negative effects of social media‐based fitspiration content (fitspo), which frequently portrays thin and toned bodies or purportedly healthy food (Tiggemann and Zaccardo 2018), as well as thinspiration content (thinspo), which often comprises content related to losing weight and showing thin bodies (Boepple and Thompson 2016). Experimental studies have found that compared to viewing neutral content, viewing fitspo results in higher negative mood and lower appearance self‐esteem (Rounds and Stutts 2021; Tiggemann and Zaccardo 2015), and viewing either fitspo or thinspo leads to increased body dissatisfaction (Dignard and Jarry 2021). Furthermore, a study with individuals with self‐reported EDs found that more frequent exposure to fitspo and thinspo was related to more frequent appearance comparisons, which were in turn associated with higher ED symptom severity (Griffiths et al. 2018).

In response, the body positivity (BoPo) movement has emerged, which promotes acceptance and love of one's body, for example by depicting a wider range of body shapes and appearances (Cohen et al. 2021). Exposure to BoPo is associated with lower levels of body dissatisfaction and fewer appearance comparisons (Fardouly et al. 2023). However, the BoPo movement has been criticized for maintaining a focus on appearance (Cohen et al. 2021). As an alternative, body neutrality (BoNeu) has emerged, which promotes a neutral, accepting attitude towards one's body (Pellizzer and Wade 2023), for example by emphasizing what the body can accomplish independently of its appearance (Alleva and Tylka 2021). BoNeu can lead to higher body satisfaction, body appreciation, and positive mood (Alleva et al. 2015; Seekis and Lawrence 2023). Comparing the trends, an experimental study by Ladwig et al. (2024) found that exposure to fitspo was linked to increased body dissatisfaction, while exposure to BoPo and BoNeu resulted in decreased body dissatisfaction and negative mood.

According to the Tripartite Influence Model (Thompson et al. 1999), these differing effects might be attributable to the mediating role of appearance comparisons (Roberts et al. 2022). In line with this suggestion, it has been demonstrated that upward appearance comparisons mediate the detrimental effects of social media in general, for example on self‐esteem (Schmuck et al. 2019), and of fitspo regarding body image concerns (Fardouly et al. 2018). Correspondingly, studies have shown that BoPo and BoNeu lead to fewer upward appearance comparisons (Rousseau 2024; Seekis and Lawrence 2023).

Aiming to understand the effects of social media contents in real‐life contexts, an increasing number of studies have used ecological momentary assessment (EMA) approaches, thus promoting external validity (Stone and Shiffman 1994). For example, Griffiths and Stefanovski (2019) asked participants to report exposure to fitspo or thinspo for 7 days at six random time points per day, and found that viewing these contents was associated with lower body satisfaction, lower positive affect, and higher negative affect. Further EMA studies have similarly suggested that fitspo is associated with negative mood and appearance comparisons (Fioravanti et al. 2021), while BoPo is related to greater body satisfaction and positive mood (Fioravanti et al. 2021; Stevens and Griffiths 2020). Several EMA studies in samples without EDs have found that higher levels of disordered eating are associated with greater exposure to thinspo and fitspo (Christensen Pacella et al. 2023), which both lead to higher disordered eating urges (Martin et al. 2023). The latter study also examined appearance comparisons as a possible mediator of the effects of fitspo and thinspo on body dissatisfaction, happiness, and disordered eating urges, but found no mediating effect (Martin et al. 2023).

Although there is a growing body of EMA research on the impact of social media contents, some questions remain. While studies have frequently examined the effects of fitspo, thinspo, BoPo, and BoNeu on mood and body image, self‐esteem has scarcely been considered, despite evidence that social media use affects self‐esteem (Schmuck et al. 2019; Vall‐Roqué et al. 2021). Moreover, there is a lack of EMA studies that specifically include women with EDs, even though this population is at a heightened risk of negative effects due to social media (Holland and Tiggemann 2016). In addition, it is important to determine the underlying mechanisms, such as upward appearance comparisons, which have rarely been examined in EMA studies, except for the study by Martin et al. (2023), who was unable to confirm a mediating role of appearance comparisons. A further limitation of the aforementioned EMA studies is that they used time‐contingent measures, meaning that participants were prompted to complete assessments at specific or random time points during the day (e.g., Griffiths and Stefanovski 2019; Martin et al. 2023; Stevens and Griffiths 2020), irrespective of whether they had actually been using social media.

Therefore, the current study aimed to conduct assessments immediately following exposure to social media. Additionally, we aimed to both validate and add to previous findings of detrimental effects of fitspo and thinspo, and beneficial effects of BoPo and BoNeu, on mood, body image, self‐esteem, and disordered eating, while especially examining potential differences between women with anorexia or bulimia nervosa (AN/BN) and women without EDs. Finally, we aimed to clarify the mediating role of upward appearance comparisons. In accordance, we formulated the following hypotheses:

1. Viewing fitspo and thinspo is associated with lower happiness, higher sadness, higher body dissatisfaction, lower body appreciation, and lower self‐esteem immediately after social media use, and with a higher degree of daily‐reported restrained eating behavior.
2. Viewing BoPo and BoNeu is associated with higher happiness, lower sadness, lower body dissatisfaction, higher body appreciation, and higher self‐esteem immediately after social media use, and with a lower degree of daily‐reported restrained eating behavior.
3. The associations of (1) and (2) differ between women with self‐reported AN/BN and women without EDs.
4. Upward appearance comparisons mediate the association between viewing fitspo, thinspo, BoPo, and BoNeu on the one hand and happiness, sadness, body dissatisfaction, body appreciation, and self‐esteem immediately after social media use on the other.

## 2 Method

### 2.1 Participants

Inclusion criteria for the present study were female gender, age ≥ 18 years, and self‐reported daily use of image‐based social media, defined as platforms on which mainly pictures, photos, reels, stories, or short videos are distributed (messaging apps were excluded). Recruitment targeted women with and without EDs. Participants were primarily recruited through social media, email distribution lists, and flyers between April 2023 and October 2024.

**FIGURE 1:**
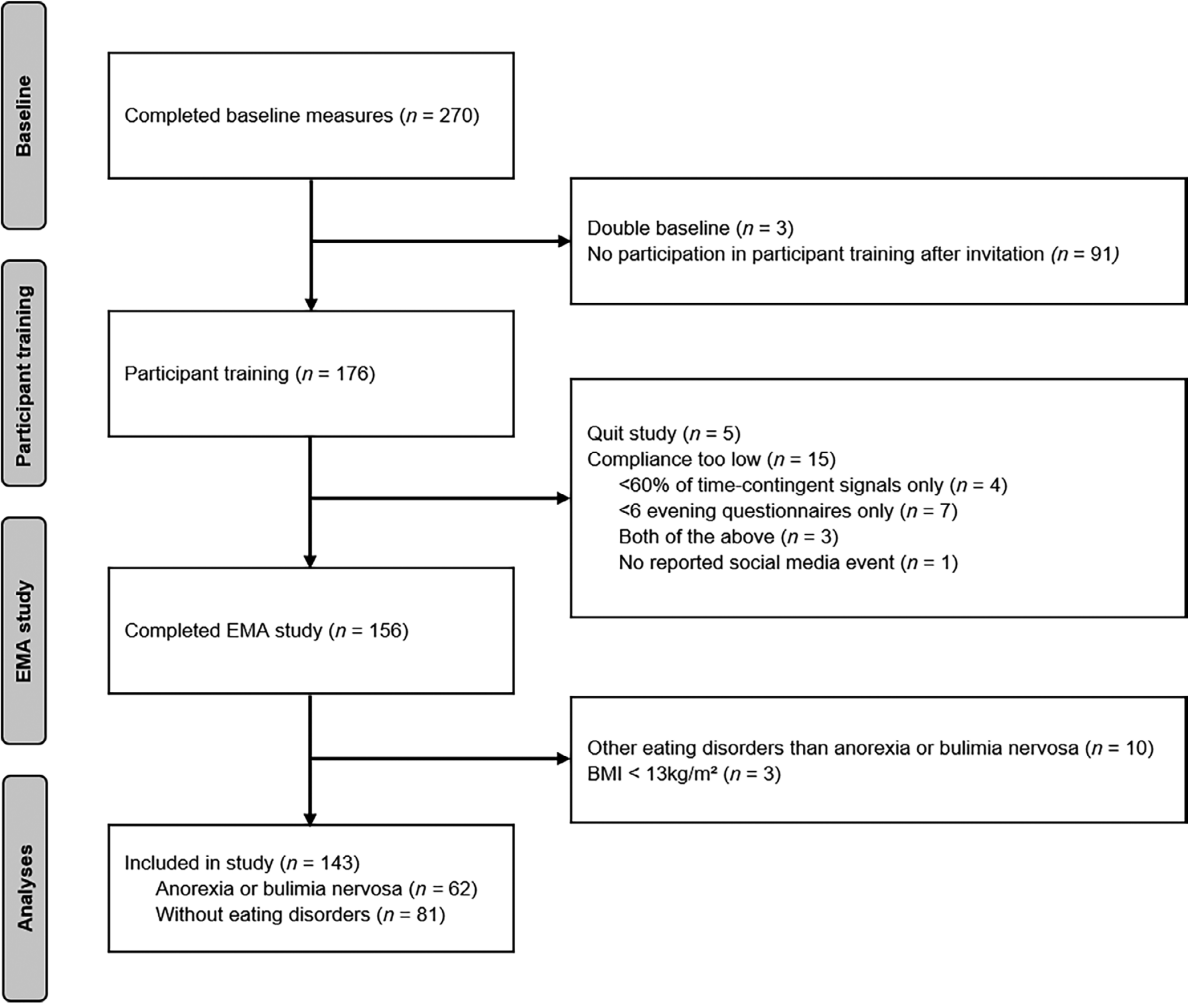
Participant flow chart.

Only participants with a body mass index (BMI) ≥ 13 kg/m^2^ were included in the analyses as in patients with AN, a lower BMI is associated with more severe cognitive impairments (Stedal et al. 2021) and a BMI below this threshold indicates extremely severe underweight with a heightened mortality risk (Schweiger and Hagenah 2018). In total, *n* = 62 women with self‐reported AN/BN (18–41 years, *M*_age_ = 23.27, SD_age_ = 4.56) and *n* = 81 women without EDs (18–53 years, *M*_age_ = 22.72, SD_age_ = 4.60) were included in the study (see flow chart in Figure 1). Of the women with self‐reported AN/BN, 96.8% indicated that the diagnosis was made by a medical practitioner or psychotherapist. Women with self‐reported AN/BN showed significantly higher ED pathology on the Eating Disorder Examination‐Questionnaire (German version: Hilbert and Tuschen‐Caffier 2016) than women without EDs (*p* < 0.001, see Supplement A). Participants' BMI ranged from 13.59 to 37.56 kg/m^2^ (*M*_BMI_ = 18.60, SD_BMI_ = 3.91) for women with AN/BN and from 16.73 to 36.33 kg/m^2^ (*M*_BMI_ = 22.32, SD_BMI_ = 3.31) for women without EDs. Further sample characteristics are presented in Table 1.

**TABLE 1:** Sample characteristics of women with self‐reported anorexia or bulimia nervosa and women without self‐reported eating disorders.

Variable	Women with AN/BN (*n* = 62)		Women without EDs (*n* = 81)	
	*n*	%	*n*	%
Eating disorder				
Anorexia nervosa	46	74.2%	None	None
Atypical anorexia nervosa	5	8.1%		
Bulimia nervosa	10	16.1%		
Atypical bulimia nervosa	1	1.6%		
Highest educational attainment				
Lower‐track secondary school	3	4.8%	1	1.2%
Medium‐track secondary school	12	19.4%	1	1.2%
Advanced technical college	4	6.5%	3	3.7%
Higher‐track secondary school	26	41.9%	69	85.2%
University of applied sciences	2	3.2%	3	3.7%
University	15	24.2%	4	4.9%
Most frequently used image‐based social media app				
Instagram	48	77.4%	57	70.4%
TikTok	11	17.7%	15	18.5%
Snapchat	2	3.2%	2	2.5%
Facebook	0	0%	1	1.2%
Pinterest	0	0%	0	0%
BeReal	0	0%	2	2.5%
Other	1	1.6%	4	4.9%
All image‐based social media apps used^a^				
Instagram	62	100%	77	95.1%
TikTok	27	43.5%	30	37.0%
Snapchat	30	48.4%	46	56.8%
Facebook	13	21.0%	10	12.3%
Pinterest	36	58.1%	45	55.6%
BeReal	15	24.2%	31	38.3%
Other	10	16.1%	9	11.1%

Abbreviations: AN/BN, self‐reported anorexia or bulimia nervosa; ED, self‐reported eating disorder.

^a^ Participants were allowed to use several image‐based social media apps, meaning that the percentage does not add up to 100.

### 2.2 EMA Measures

#### 2.2.1 Exposure to Social Media Contents

Directly after using social media, participants reported exposure to fitspo, thinspo, BoPo, or BoNeu, the social media platforms visited, and the amount of time spent on these platforms.

#### 2.2.2 State Mood

State mood was assessed using the question “How are you feeling right now, in this moment?” rated on visual analogue scales (VAS) from 0 = not at all to 100 = extremely for the items “happy” and “sad”, derived from the Positive and Negative Affect Schedule—Expanded Form (PANAS‐X; German version: Grühn et al. 2010).

#### 2.2.3 State Body Dissatisfaction

State body dissatisfaction was assessed using the statement “Right now, in this moment, I am dissatisfied with my body”, rated on a VAS ranging from 0 = not at all to 100 = extremely.

#### 2.2.4 State Body Appreciation

To assess state body appreciation, we used an adapted item from the Body Appreciation Scale 2 (BAS‐2; German version: Behrend and Warschburger 2022): “Right now, in this moment, I have a positive attitude towards my body”, rated on a VAS ranging from 0 = not at all to 100 = extremely.

#### 2.2.5 State Self‐Esteem

State self‐esteem was assessed using the German Single‐Item Self‐Esteem scale (G‐SISE; Brailovskaia and Margraf 2020). The item “I have high self‐esteem” was rated on a 5‐point Likert scale ranging from 1 = not at all true of me to 5 = very true of me.

#### 2.2.6 State Upward Appearance Comparisons

To capture state upward appearance comparisons on social media, we adapted one item from the Upward Physical Appearance Comparison Scale (UPACS; German version: Schönhals et al. 2024): “I compared my body with people on social media who have a better body than me”, rated on a 5‐point Likert scale ranging from 1 = strongly disagree to 5 = strongly agree.

#### 2.2.7 Restrained Eating Behaviors

To assess the occurrence of restrained eating behaviors, we adapted five items from the Restraint subscale (restriction of food intake, not eating for at least 8 h, excluding liked foods, following food‐related rules, desire for an empty stomach) of the Eating Disorder Examination‐Questionnaire (EDE‐Q; German version: Hilbert and Tuschen‐Caffier 2016), with participants indicating whether they had shown the respective behaviors during the day using a dichotomous response format (yes/no).

### 2.3 Procedure

Participants provided sociodemographic data and completed baseline measures (see Supplement A) online via LimeSurvey (Version 6.4.2 + 240115). Subsequently, they were invited to attend a participant training session, either online or face‐to‐face. The training included descriptions of fitspo, thinspo, BoPo, and BoNeu, as well as instructions for following the EMA protocol, which was administered using the app *movisensXS* (Versions 1.5.23 to 1.6.3, movisens GmbH, Karlsruhe, Germany). Participants could use their own Android smartphone or borrow a study smartphone (Motorola Moto G42). On the evening following the training as well as the day after, a test trial was administered to ensure that participants understood the EMA protocol. One day later, participants began the 7‐day EMA. They were instructed to self‐initiate the social media questionnaire directly after social media use (event‐contingent measure) in order to report exposure to social media contents, state mood, body dissatisfaction, body appreciation, appearance comparisons on social media, and self‐esteem. Additionally, participants received four semi‐random prompts per day, between 10:00 am and 10:00 pm (time‐contingent measure), to assess state mood, body dissatisfaction, body appreciation, and self‐esteem. Every evening, participants completed a questionnaire assessing restrained eating behavior on that day. Participants were reimbursed with course credit or vouchers. The study was approved by the Ethics committee of Osnabrück University.

### 2.4 Data Analysis

Participants who provided at least one event‐contingent measure, 60% of time‐contingent measures, and six evening questionnaires were included in the data analysis in order to consider only participants who adhered to the study protocol. These *n* = 143 participants reported *M* = 15.92 (SD = 9.20) event‐contingent measures, *M* = 25.27 (SD = 2.82) time‐contingent measures, and *M* = 6.86 (SD = 0.37) evening questionnaires. Further descriptive statistics can be derived from Table 2. Age, diagnosis, BMI, and ED pathology did not predict the number of time‐contingent measures reported (all *p* > 0.05). To impute missing values regarding the time‐contingent measures and the evening questionnaires, random forest imputation was conducted using the R package *missForest* (Stekhoven and Bühlmann 2012). For all imputations, the normalized root mean square error of approximation (NRMSEA; Chai and Draxler 2014) values remained under 0.001, indicating that the average imputation error was estimated to fall below 0.001 standard deviations, while for categorical variables, less than 6% of values were falsely classified. Little's MCAR (missing completely at random) test indicated that the pattern of missingness across the EMA variables was not consistent with MCAR (*χ* ^2^ (14) = 37.56, *p* = 0.0006) based on five missing data patterns, suggesting that the data were not missing completely at random. However, as the *missForest* imputation procedure is appropriate under both MCAR and missing at random (MAR) assumptions (Stekhoven and Bühlmann 2012), the imputed dataset remains valid under MAR assumptions.

**TABLE 2:** Descriptive statistics of the event‐contingent measures.

Variable	Women with AN/BN (*n* = 62)		Women without EDs (*n* = 81)	
	*M*	SD	*M*	SD
Social media events	17.66	8.38	14.58	9.61
Average time on social media	24.97	18.09	22.59	16.79
Percentage of social media episodes^a^ in which participants viewed…				
Fitspiration	52.7%	0.29	33.8%	0.27
Thinspiration	31.6%	0.27	12.6%	0.19
Body positivity	25.4%	0.23	16.5%	0.20
Body neutrality	26.6%	0.23	14.0%	0.19
Only fitspiration/thinspiration^b^	31.5%	0.24	25.6%	0.23
Only body positivity/neutrality^c^	10.8%	0.12	12.8%	0.15
Mix of the contents^d^	31.5%	0.24	12.4%	0.17
None of the contents	26.2%	0.25	49.3%	0.27

*Note*: Social media events = reported social media events during the seven‐day study period. Average time on social media = Mean of the average time spent on social media per social media episode per participant in minutes.

Abbreviations: AN/BN, self‐reported anorexia or bulimia nervosa; ED, eating disorder.

^a^ Average of the individual percentages per participant.

^b^ Only fitspiration, only thinspiration, or only the combination of fitspiration and thinspiration (i.e., not in the same session as body positivity/body neutrality).

^c^ Only body positivity, only body neutrality, or only the combination of body positivity and body neutrality (i.e., not in the same session as fitspiration/thinspiration).

^d^ Fitspiration or thinspiration were viewed together with body positivity and/or body neutrality.

To test the hypotheses, two‐level Bayesian multilevel modeling was performed using the *brms* package (Bürkner 2018) in R (version 4.4.1). Level 1 consisted of the individual time points at which participants answered the EMA questionnaires. These were nested within the participants, which comprised level 2. For hypotheses (1) to (3), the analyses were performed for each immediate outcome, that is, sadness, happiness, body dissatisfaction, body appreciation, and self‐esteem, as well as for the Restraint scale of the EDE‐Q. For the immediate outcomes, event‐contingent as well as time‐contingent measures were considered. The latter were included in order to account for fluctuations in the outcomes over the course of the day. The group factor “diagnosis” was included, with self‐reported AN/BN coded as “1” and no ED coded as “2”. For daily restrained eating behavior, only event‐contingent measures were considered in order to compute the daily frequency of viewing the four contents as the predictor. The general model is as follows:

$$\begin{array}{c} {\text{outcome}}_{\mathrm{ij}}=\left(\gamma_{00}+u_{0j}\right)+\gamma_{01}\times {\bar{\text{fitspiration}}}_{1j}\\ \;+\gamma_{02}\times {\bar{\text{thinspiration}}}_{2j}\\ \;+\gamma_{03}\times {\bar{\text{body}\_\text{positivity}}}_{3j}\\ \;+\gamma_{04}\times {\bar{\text{body}\_\text{neutrality}}}_{4j}\\ \;+\gamma_{10}\times \text{fitspiratio}n_{\mathrm{ij}}+\gamma_{20}\times \text{thinspiratio}n_{\mathrm{ij}}\\ \;+\gamma_{30}\times \text{body}\_\text{positivit}y_{\mathrm{ij}}+\gamma_{40}\\ \;\times \text{body}\_\text{neutralit}y_{\mathrm{ij}}+\gamma_{50}\times \text{diagnosi}s_{0j}\\ \;+\gamma_{60}\times \text{fitspiration}:\text{diagnosi}s_{\mathrm{ij}}\\ \;+\gamma_{70}\times \text{thinspiration}:\text{diagnosi}s_{\mathrm{ij}}\\ \;+\gamma_{80}\times \text{body}\_\text{positivity}:\text{diagnosi}s_{\mathrm{ij}}\\ \;+\gamma_{90}\times \text{body}\_\text{neutrality}:\text{diagnosi}s_{\mathrm{ij}}+e_{\mathrm{ij}} \end{array}$$

The index *i* represents the individual measurement time point on level 1 and *j* the respective participant on level 2. The model contains a random intercept ($u_{0j}$) on level 2 to account for interindividual outcome differences. The time‐dependent predictors (e.g., fitspo) were disaggregated into a person‐specific mean (${\bar{\text{fitspiration}}}_{1j}$) and a person‐centered ($\text{fitspiratio}n_{\mathrm{ij}}$) variable. Since the hypotheses were concerned with effects on a within‐person level, we report effect sizes and *p*‐values for main and interaction effects only for the within‐person level. The within‐person estimates report the effect of viewing the content versus not viewing the content.

For hypothesis (4), upward appearance comparisons on social media were included as a mediator of the association between each content viewed and sadness, happiness, body dissatisfaction, body appreciation, and self‐esteem. The mediation analyses were conducted for the event‐contingent measures only, and did not include the group factor diagnosis. Again, a within‐ and between‐person disaggregation was conducted for content viewed and for upward comparison. The general model formula is as follows:

$$\begin{array}{c} \left(4a\right)\;{\text{upward}}_{\mathrm{ij}}=\left(\alpha_{00}+u_{0j}\right)+\alpha_{01}\times {\bar{\text{fitspiration}}}_{1j}\\ \;+\alpha_{02}\times {\bar{\text{thinspiration}}}_{2j}+\alpha_{03}\\ \;\times {\bar{\text{body}\_\text{positivity}}}_{3j}\\ \;+\alpha_{04}\times {\bar{\text{body}\_\text{neutrality}}}_{4j}\\ \;+\alpha_{10}\times \text{fitspiratio}n_{\mathrm{ij}}\\ \;+\alpha_{20}\times \text{thinspiratio}n_{\mathrm{ij}}+\alpha_{30}\\ \;\times \text{body}\_\text{positivit}y_{\mathrm{ij}}+\alpha_{40}\\ \;\times \text{body}\_\text{neutralit}y_{\mathrm{ij}}+e_{\mathrm{ij}} \end{array}$$

$$\begin{array}{c} \left(4b\right)\;\;{\text{outcome}}_{\mathrm{ij}}=\left(\beta_{00}+u_{0j}\right)+\beta_{01}\times {\bar{\text{fitspiration}}}_{1j}\\ \;+\beta_{02}\times {\bar{\text{thinspiration}}}_{2j}+\beta_{03}\\ \;\times {\bar{\text{body}\_\text{positivity}}}_{3j}\\ \;+\beta_{04}\times {\bar{\text{body}\_\text{neutrality}}}_{4j}\\ \;+\beta_{05}\times {\bar{\text{upward}}}_{5j}+\beta_{10}\times \text{fitspiratio}n_{\mathrm{ij}}\\ \;+\beta_{20}\times \text{thinspiratio}n_{\mathrm{ij}}+\beta_{30}\\ \;\times \text{body}\_\text{positivit}y_{\mathrm{ij}}+\beta_{40}\\ \;\times \text{body}\_\text{neutralit}y_{\mathrm{ij}}+\beta_{50}\\ \;\times \text{upwar}d_{\mathrm{ij}}+e_{\mathrm{ij}} \end{array}$$

While (4a) models the prediction of the mediator by content viewed, (4b) represents the effect of the mediator on outcome while controlling for the effect of the contents. The time‐dependent predictors were disaggregated into a person‐specific mean (e.g., ${\bar{\text{upward}}}_{5j}$) and a person‐centered (e.g., $\text{upwar}d_{\mathrm{ij}}$) variable. The mediation effect was calculated on a within‐person level by multiplying the respective person‐centered effect on *upward* (i.e., $\alpha_{10}–\alpha_{40}$) by the person‐centered effect of *upward* on outcome (i.e., $\beta_{50}$).

To test hypotheses (1) and (2), that is, the main effects, we conducted one‐tailed tests, while to test hypotheses (3) and (4), that is, the interaction and mediation effects, we conducted two‐tailed tests with an alpha level of 0.05. To correct for multiple testing across each of the outcomes, we applied Benjamini‐Hochberg correction with a false discovery rate of 0.05. There were no violations of the assumptions of linearity or normality of error distributions across the outcomes. We found variance inflation (variance inflation factor > 5) for the interactions between person‐specific means and diagnosis. Since it is possible to model within‐person effects without including between‐person effects (e.g., Benitez et al. 2019) and the between‐person effects were not relevant for our hypotheses, these were removed. Beyond this, no multicollinearity was found. Effect sizes were calculated by dividing the respective fixed effect by the square root of the sum of error variance and random intercept variance (Westfall et al. 2014), which can be roughly interpreted according to Cohen's *d* (0.2 = small, 0.5 = moderate, 0.8 = large; Cohen 1988). Further analyses concerning baseline measures, social media use, compliance, and an exemplary post hoc power analysis are presented in Supplement A.

## 3 Results

Hypothesis (1), regarding associations between exposure to fitspo or thinspo and detrimental outcomes, was fully confirmed for exposure to thinspo and partially confirmed for exposure to fitspo for the immediate outcomes, but not for daily‐reported restrained eating behavior (see Table 3). The main effects of exposure to fitspo as well as thinspo were significant for lower happiness, higher body dissatisfaction, and lower body appreciation (all *p* < 0.01, 0.13 ≤ |*d*| ≤ 0.26). Only the main effects of exposure to thinspo, but not to fitspo (both *p* > 0.05), were significant for higher sadness and lower self‐esteem (both *p* < 0.05, 0.15 ≤ |*d*| ≤ 0.32). There were no main effects for the Restraint subscale of the EDE‐Q (all *p* > 0.05).

**TABLE 3:** Main effects and interaction effects of the content viewed (i.e., fitspiration, thinspiration, body positivity, and body neutrality) and eating disorder diagnosis on happiness, sadness, body dissatisfaction, body appreciation, and self‐esteem immediately after using social media, and daily‐reported restrained eating behavior.

Model	Estimate	Est. Error	*p* ^a^	*p* adj.^b^	Effect size *d* (CI)^c^
Happiness					
Main effects					
Diagnosis	25.07	2.93			1.14 (0.86, 1.40)
Fitspiration	−2.92	0.99	0.002	0.005	−0.13 (−0.22, −0.05)
Thinspiration	−4.54	1.13	< 0.001	< 0.001	−0.21 (−0.31, −0.11)
Body positivity	−0.32	1.14	0.612	0.874	−0.02 (−0.12, 0.09)
Body neutrality	0.95	1.15	0.205	0.352	0.04 (−0.06, 0.15)
Interactions					
Diagnosis × fitspiration	−0.33	1.43	0.809	0.883	−0.02 (−0.14, 0.11)
Diagnosis × thinspiration	0.66	2.00	0.737	0.883	0.03 (−0.15, 0.21)
Diagnosis × body positivity	−0.03	1.75	0.990	0.990	0.00 (−0.16, 0.15)
Diagnosis × body neutrality	−0.83	1.87	0.656	0.874	−0.04 (−0.21, 0.13)
Sadness					
Main effects					
Diagnosis	−23.69	2.92			−1.06 (−1.32, −0.80)
Fitspiration	1.17	0.99	0.123	0.164	0.05 (−0.04, 0.14)
Thinspiration	7.19	1.12	< 0.001	< 0.001	0.32 (0.22, 0.42)
Body positivity	0.82	1.12	0.766	0.836	0.04 (−0.06, 0.14)
Body neutrality	−2.06	1.14	0.035	0.083	−0.09 (−0.19, 0.01)
Interactions					
Diagnosis × fitspiration	−0.02	1.44	0.980	0.980	0.00 (−0.13, 0.12)
Diagnosis × thinspiration	−4.61	1.96	0.018	0.054	−0.21 (−0.38, −0.03)
Diagnosis × body positivity	−0.95	1.74	0.577	0.693	−0.04 (−0.20, 0.11)
Diagnosis × body neutrality	2.88	1.85	0.115	0.164	0.13 (−0.03, 0.29)
Body dissatisfaction					
Main effects					
Diagnosis	−32.71	3.37			−1.48 (−1.80, −1.15)
Fitspiration	2.90	0.91	< 0.001	0.002	0.13 (0.05, 0.22)
Thinspiration	5.79	1.01	< 0.001	< 0.001	0.26 (0.17, 0.36)
Body positivity	−0.30	1.02	0.385	0.577	−0.01 (−0.10, 0.08)
Body neutrality	−1.41	1.04	0.085	0.146	−0.06 (−0.16, 0.03)
Interactions					
Diagnosis × fitspiration	0.36	1.32	0.781	0.852	0.02 (−0.10, 0.13)
Diagnosis × thinspiration	−0.60	1.75	0.738	0.852	−0.03 (−0.19, 0.13)
Diagnosis × body positivity	−1.06	1.57	0.500	0.667	−0.05 (−0.19, 0.09)
Diagnosis × body neutrality	−0.20	1.70	0.904	0.904	−0.01 (−0.16, 0.14)
Body appreciation					
Main effects					
Diagnosis	32.89	3.16			1.55 (1.23, 1.86)
Fitspiration	−3.67	0.81	< 0.001	< 0.001	−0.17 (−0.25, −0.10)
Thinspiration	−4.00	0.91	< 0.001	< 0.001	−0.19 (−0.28, −0.10)
Body positivity	0.46	0.92	0.311	0.414	0.02 (−0.06, 0.11)
Body neutrality	2.57	0.95	0.004	0.009	0.12 (0.03, 0.21)
Interactions					
Diagnosis × fitspiration	0.82	1.17	0.485	0.582	0.04 (−0.07, 0.15)
Diagnosis × thinspiration	0.57	1.61	0.726	0.792	0.03 (−0.12, 0.18)
Diagnosis × body positivity	−0.19	1.41	0.884	0.884	−0.01 (−0.14, 0.12)
Diagnosis × body neutrality	−1.89	1.56	0.222	0.333	−0.09 (−0.23, 0.06)
Self‐esteem					
Main effects					
Diagnosis	0.40	0.15			0.33 (0.08, 0.57)
Fitspiration	−0.10	0.06	0.041	0.099	−0.09 (−0.18, 0.01)
Thinspiration	−0.18	0.07	0.003	0.013	−0.15 (−0.25, −0.04)
Body positivity	0.08	0.07	0.131	0.175	0.06 (−0.05, 0.17)
Body neutrality	0.01	0.07	0.470	0.513	0.00 (−0.10, 0.11)
Interactions					
Diagnosis × fitspiration	0.09	0.09	0.297	0.356	0.07 (−0.07, 0.21)
Diagnosis × thinspiration	0.31	0.12	0.010	0.031	0.26 (0.06, 0.45)
Diagnosis × body positivity	0.18	0.10	0.090	0.134	0.14 (−0.02, 0.31)
Diagnosis × body neutrality	0.01	0.11	0.911	0.911	0.01 (−0.16, 0.19)
EDE‐Q restraint					
Main effects					
Diagnosis	2.98	0.24			1.74 (1.44, 2.03)
Fitspiration	−0.05	0.08	0.719	0.744	−0.03 (−0.12, 0.07)
Thinspiration	0.11	0.13	0.194	0.628	0.07 (−0.09, 0.22)
Body positivity	−0.04	0.10	0.346	0.628	−0.02 (−0.14, 0.10)
Body neutrality	0.08	0.12	0.744	0.744	0.05 (−0.10, 0.19)
Interactions					
Diagnosis × fitspiration	0.12	0.12	0.311	0.628	0.07 (−0.06, 0.21)
Diagnosis × thinspiration	−0.12	0.17	0.471	0.628	−0.07 (−0.27, 0.12)
Diagnosis × body positivity	0.13	0.14	0.391	0.628	0.07 (−0.09, 0.24)
Diagnosis × body neutrality	−0.13	0.16	0.406	0.628	−0.08 (−0.26, 0.10)

*Note*: Within‐person effects except for diagnosis (anorexia/bulimia nervosa vs. no eating disorder), which is only reported for completeness and was not explicitly tested.

Abbreviations: CI, confidence interval; EDE‐Q restraint, daily‐reported Restraint subscale of the Eating Disorder Examination‐Questionnaire.

^a^ Unadjusted, *p*‐value is one‐tailed for the main effects and two‐tailed for the interaction effects.

^b^ Adjusted with Benjamini‐Hochberg correction across each outcome (Immediate outcomes: 12 tests—four main effects, four interaction effects, four mediation effects from Table 4 per outcome. EDE‐Q: 8 tests—four main effects, four interaction effects), *p*‐value is one‐tailed for the main effects and two‐tailed for the interaction effects.

^c^ CI is 90% for the main effects and 95% for the interaction effects.

Overall, Hypothesis (2), regarding associations of BoPo and BoNeu with beneficial outcomes, was not supported. There were no main effects of exposure to the two contents on happiness, sadness, body dissatisfaction, or self‐esteem immediately after social media use, or on the Restraint subscale of the EDE‐Q (all *p* > 0.05, see Table 3). Only the main effect of exposure to BoNeu, but not to BoPo (*p* = 0.41), was significant for higher body appreciation (*p* = 0.009, *d* = 0.12).

Hypothesis (3), regarding differences in the associations of exposure to the four contents with the outcomes between women with self‐reported AN/BN and women without EDs, was only supported for one interaction (all other *p* > 0.05, see Table 3). The interaction effect of diagnosis × thinspo was significant for self‐esteem (*p* = 0.03, *d* = 0.26): Compared to women without EDs, women with AN/BN showed a stronger reduction in self‐esteem after viewing thinspo.

Hypothesis (4), regarding the mediation by upward appearance comparisons on social media, was supported for fitspo and thinspo but not for BoPo and BoNeu (see Table 4). Upward appearance comparisons during social media use significantly mediated the associations of fitspo and thinspo (all *p* < 0.001, 0.06 ≤ |*d*| ≤ 0.09), but not of BoPo and BoNeu (all *p* > 0.05), with sadness, happiness, body dissatisfaction, body appreciation, and self‐esteem immediately after social media use. The full mediation models are provided in Supplement B.

**TABLE 4:** Mediating effects of upward appearance comparisons during social media exposure for the effects of fitspiration, thinspiration, body positivity, and body neutrality on happiness, sadness, body dissatisfaction, body appreciation, and self‐esteem immediately after using social media.

Model	Estimate	Est. Error	*p* (two‐tailed)	*p* adj.^a^ (two‐tailed)	Effect size *d* (95% CI)	% mediation
Happiness						
Fitspiration	−1.28	0.23	< 0.001	< 0.001	−0.07 (−0.09, −0.04)	44.4%
Thinspiration	−1.38	0.26	< 0.001	< 0.001	−0.07 (−0.10, −0.05)	36.6%
Body positivity	−0.21	0.12	0.065	0.144	−0.01 (−0.02, 0.00)	34.2%
Body neutrality	−0.22	0.13	0.072	0.144	−0.01 (−0.03, 0.00)	12.5%
Sadness						
Fitspiration	1.41	0.24	< 0.001	< 0.001	0.07 (0.05, 0.10)	82.7%^b^
Thinspiration	1.52	0.27	< 0.001	< 0.001	0.08 (0.05, 0.11)	25.4%
Body positivity	0.23	0.13	0.069	0.117	0.01 (0.00, 0.03)	24.1%
Body neutrality	0.24	0.14	0.064	0.117	0.01 (0.00, 0.03)	20.0%
Body dissatisfaction						
Fitspiration	1.60	0.24	< 0.001	< 0.001	0.08 (0.06, 0.10)	47.0%
Thinspiration	1.72	0.27	< 0.001	< 0.001	0.09 (0.06, 0.12)	28.3%
Body positivity	0.26	0.15	0.060	0.144	0.01 (0.00, 0.03)	19.5%^b^
Body neutrality	0.28	0.16	0.072	0.144	0.01 (0.00, 0.03)	18.4%^b^
Body appreciation						
Fitspiration	−1.26	0.20	< 0.001	< 0.001	−0.06 (−0.09, −0.04)	33.6%
Thinspiration	−1.36	0.23	< 0.001	< 0.001	−0.07 (−0.09, −0.05)	36.1%
Body positivity	−0.21	0.12	0.075	0.129	−0.01 (−0.02, 0.00)	19.2%
Body neutrality	−0.22	0.13	0.068	0.129	−0.01 (−0.02, 0.00)	10.2%
Self‐esteem						
Fitspiration	−0.06	0.01	< 0.001	< 0.001	−0.07 (−0.09, −0.05)	70.6%
Thinspiration	−0.07	0.01	< 0.001	< 0.001	−0.07 (−0.10, −0.05)	49.5%
Body positivity	−0.01	0.01	0.056	0.113	−0.01 (−0.02, 0.00)	16.0%^b^
Body neutrality	−0.01	0.01	0.068	0.116	−0.01 (−0.03, 0.00)	97.8%

*Note*: The table shows the indirect pathway of the mediation models, see Supplement B. The independent variable is the content viewed (i.e., fitspiration, thinspiration, body positivity, body neutrality), the mediator is upward appearance comparisons on social media, and the dependent variables are happiness, sadness, body dissatisfaction, body appreciation, and self‐esteem. The table shows within‐person effects.

Abbreviation: CI, confidence interval.

^a^ Adjusted with Benjamini‐Hochberg correction across each outcome (12 tests—four effects and four interaction effects from Table 3, four mediation effects per outcome).

^b^ As the mediation effect is a suppression effect, the total effect was calculated by adding the absolute values of the direct effect and the indirect effect.

## 4 Discussion

The present study examined associations of exposure to fitspo, thinspo, BoPo, and BoNeu with mood, body image, self‐esteem, and disordered eating behavior in women with self‐reported AN/BN and women without EDs who use social media daily, utilizing an EMA design in everyday life.

In line with our hypothesis, exposure to fitspo and thinspo was associated with lower happiness, higher body dissatisfaction, and lower body appreciation directly after using social media as compared to non‐exposure to these contents. These findings support previous research that reported detrimental effects of these types of content (e.g., Dignard and Jarry 2021; Fioravanti et al. 2021; Griffiths and Stefanovski 2019; Ladwig et al. 2024). Only exposure to thinspo was associated with higher sadness and lower self‐esteem. Importantly, the association of thinspo with lower general self‐esteem, which is not explicitly appearance‐related, indicates that the influence of thinspo might reach beyond body image alone, while exposure to fitspo might only be associated with appearance‐related self‐esteem (Tiggemann and Zaccardo 2015), but not with self‐esteem in general. Overall, compared to fitspo, viewing thinspo was more consistently related to a deterioration of the outcomes, with consistently larger effect sizes, which supports previous studies reporting thinspo to be the more harmful type of content (e.g., Griffiths and Stefanovski 2019).

The finding that fitspo was not associated with higher sadness is in contrast to previous studies that found strong associations between exposure to fitspo and negative affect (Jerónimo and Carraça 2022), but is in line with the experimental study by Ladwig et al. (2024), who found no such association. The lack of association in the present study might be due to the fact that we only assessed sadness, whereas viewing fitspo might exert a stronger impact on other negative emotions, such as guilt or shame, which are associated with body dissatisfaction and EDs (Blythin et al. 2020).

Furthermore, while the lack of association between viewing fitspo or thinspo and daily‐reported restrained eating behavior contradicts previous research regarding disordered eating behaviors (Griffiths et al. 2018; Martin et al. 2023), it corresponds to an experimental EMA study by Krug et al. (2020), who found no effects of viewing fitspo on disordered eating behaviors. It might be speculated that risk factors outside of these social media contents, such as pressures from family, peers, and traditional media (Thompson et al. 1999), influence disordered eating behavior to a greater extent.

In contrast to our second hypothesis, there were almost no associations of BoPo and BoNeu with beneficial changes in mood, body image, or daily‐reported restrained eating behavior in everyday life, contradicting previous research that demonstrated a positive effect of BoPo and BoNeu on mood and body satisfaction (Fioravanti et al. 2021; Ladwig et al. 2024; Stevens and Griffiths 2020). Only exposure to BoNeu was associated with higher body appreciation, which fits in well with such content's goal of promoting a more accepting attitude towards one's body (Pellizzer and Wade 2023) and might indicate that exposure to BoNeu could have more beneficial effects than BoPo, as the latter still emphasizes appearance (Cohen et al. 2021). A potential explanation for the lack of associations for most outcomes could be that the participants in our study were descriptively more likely to view BoPo and BoNeu in the same session as fitspo and thinspo rather than separately (see Supplement A). As such, the influence of fitspo and thinspo might have masked the positive effects of BoPo and BoNeu. In line with this suggestion, Hepburn and Mulgrew (2023) found that body appreciation or functionality messages did not buffer the negative effects of fitspo images, potentially indicating that the detrimental effects were too strong to counteract.

Our third hypothesis, that all of the examined associations would differ between women with self‐reported AN/BN and women without EDs, was only supported for one interaction. Specifically, women with AN/BN showed a stronger deterioration in self‐esteem after viewing thinspo compared to women without EDs. The diagnostic criteria for both AN and BN include that self‐evaluation is heavily influenced by the evaluation of body shape and weight (American Psychiatric Association 2022), suggesting that in women with AN/BN, self‐esteem might be especially negatively impacted by thinspo, whereas women without EDs may base their self‐esteem on a broader array of dimensions, such as social contacts (Rentzsch et al. 2016). Apart from this finding, associations of viewing the four contents with happiness, sadness, body dissatisfaction, and body appreciation did not differ between the two groups. These results add to the experimental study by Ladwig et al. (2024), which similarly reported no differences between women with and without EDs regarding the effects of exposure to fitspo, BoPo, and BoNeu on mood and body dissatisfaction. The results of the present study suggest that these experimental findings might be generalized to everyday life. The lack of differences might be attributable to the fact that social comparisons are evoked in women with AN/BN and women without EDs alike, indicating that both groups are affected by appearance‐related social media content (Ladwig et al. 2024).

Nonetheless, in the present study, women with self‐reported AN/BN reported viewing fitspo and thinspo descriptively more often than did women without EDs (see Supplement A), suggesting that women with AN/BN are more frequently confronted with this harmful content. In this vein, a previous EMA study showed that certain aspects of ED pathology predict higher exposure to fitspo and/or thinspo (Christensen Pacella et al. 2023). Given their greater exposure to such content, it is important to focus on women with EDs concerning the prevention of harmful effects of social media use.

Partially confirming our fourth hypothesis, upward appearance comparisons mediated all associations between viewing fitspo and thinspo on the one hand and sadness, happiness, body dissatisfaction, body appreciation, and self‐esteem immediately after social media use on the other. Therefore, the present results support previous cross‐sectional studies highlighting that upward appearance comparisons on social media play a major role in well‐being, self‐esteem, and body image (Fardouly et al. 2017; Schmuck et al. 2019) and emphasize that the influence of such comparisons may be particularly salient in everyday life in the case of fitspo and thinspo. In contrast to our hypothesis, we found no mediations of the associations of BoPo and BoNeu with the outcomes by upward comparisons. This contradicts previous studies (Rousseau 2024; Seekis and Lawrence 2023) but might be explained by the overall lack of associations found for these two contents and that, again, these contents were often viewed simultaneously with thinspo and/or fitspo, which might have evoked more upward appearance comparisons.

Some limitations need to be addressed when interpreting the present findings. First, the event‐contingent design of the study entails a risk of under‐reporting. However, 73.2% of the participants reported no or only one missing event‐contingent measure during the EMA period (see Supplement A). Second, participants reported whether they had viewed the contents, but not for how long or how often. As such, if some contents were seen for longer or more frequently than others, this might have led to a distortion of the effects. Furthermore, even though a training session was provided to ensure that participants were able to recognize the contents of interest, participants might differ in reporting whether they categorize content as, for example, fitspo, even if they viewed identical content. Moreover, this conscious act of categorization might have influenced the participants' subsequent ratings on mood, body image, and self‐esteem. Therefore, future research should track social media usage in the background, recording the actual contents viewed, in order to more objectively assess what participants see and for how long, and to prevent participants from consciously categorizing the content. Third, to reduce participant burden, all constructs were assessed using only one item. With the exception of the G‐SISE, all of the items were adapted from more comprehensive and often trait‐like questionnaires, and have not been validated as single‐item measures. As the G‐SISE item was not adapted to refer to the time point “right now”, it is possible that it did not capture state self‐esteem. Nonetheless, there were fluctuations within the item, indicating that it did not measure a stable trait, allowing us to detect a significant association in terms of lower self‐esteem after viewing thinspiration content. Furthermore, the measurements all relied on self‐report from the participants, which might limit the validity of the results. Fourth, as the exemplary post hoc power analysis (see Supplement A) suggests, the study might have been underpowered to detect interaction effects. Therefore, it would be advisable to replicate the findings with a larger sample size. Fifth, the educational level differed between women with self‐reported AN/BN and women without EDs, which might confound conclusions regarding differences between the two groups. Sixth, as the diagnoses were self‐reported, there is a risk that participants reported presumed or false diagnoses. Nonetheless, women with self‐reported AN/BN did show significantly higher scores on ED pathology than women without EDs, and 96.9% of the women with self‐reported AN/BN indicated that a medical practitioner or psychotherapist diagnosed the ED. Moreover, the group of women without EDs also included participants with a BMI < 18.5 kg/m^2^. Therefore, even though a low BMI is not the only criterion for AN, there might still be participants with undetected AN in this group. Lastly, women with AN and BN were analyzed within one group, and it is possible that women with these two disorders react differently to social media content. Despite these limitations, to the best of our knowledge, our study is the first to examine the associations of fitspo, thinspo, BoPo, and BoNeu with the various outcomes using an event‐contingent design and including women with self‐reported AN/BN.

Overall, the present findings underline the possible detrimental influence of fitspo and especially thinspo on mood, body image, and self‐esteem, while demonstrating that current counter‐movements, that is, BoPo and BoNeu, might only exert a limited beneficial effect on these outcomes in everyday life, with BoNeu showing a positive association with body appreciation. Furthermore, our findings emphasize the mediating role of upward appearance comparisons on body image, mood, and self‐esteem when viewing fitspo and thinspo, highlighting the need for specific prevention and intervention strategies to tackle the impact of such appearance comparisons. Future ED treatment programs should include psychoeducation about the detrimental effects of fitspo and thinspo and about how to better navigate content on social media.

## Author Contributions

**Kristine Schönhals:** conceptualization, formal analysis, investigation, methodology, project administration, visualization, writing – original draft. **Christopher Lalk:** formal analysis, writing – review and editing. **Silja Vocks:** supervision, writing – review and editing.

## Funding

The authors have nothing to report.

## Conflicts of Interest

The authors declare no conflicts of interest.

## Supporting information


**Data S1:** Supplement A.

**Data S2:** Supplement B.

## Data Availability

The data that support the findings of this study are available on request from the corresponding author.
