# Supplementary material for: Fitspiration, Thinspiration, Body Positivity, and Body Neutrality Contents on Image‐Based Social Media: Associations With Body Image, Mood, Self‐Esteem, and Disordered Eating Behavior in Women With and Without Self‐Reported Eating Disorders—An Ecological Momentary Assessment Study
Source: Int J Eat Disord. 2026 Jan 8;59(4):790–802. doi: 10.1002/eat.70027 (PMC13058400; doi:10.1002/eat.70027)
Supplement: Supplementary file 1 — Data S1: Supplement A. [file EAT-59-790-s002.docx]

**Supplement A**

**Exemplary post-hoc power analysis**

An exemplary post-hoc power analysis was calculated for the main and interaction effect of fitspiration and fitspiration × diagnosis on body dissatisfaction based on a frequentist mixed model using the R package *simr* (Green & MacLeod, 2016). We found a power of 88.7 % for the one-sided testing of the main effect, and a power of 6.6% for the two-sided testing of the interaction effect.

**Additional information regarding social media use during the data assessment period**

In the time-contingent measures, participants were asked to indicate whether they had forgotten to assess social media use since the last time-contingent measure. The majority of participants reported never forgetting (*n*= 74, 51.7%) or forgetting only once (*n* = 30, 21.0%). The remainder indicated forgetting to assess social media use at least twice and at most six times (*n* = 39, 27.3%).

**Additional information regarding baseline measures**

***Eating Disorder Examination Questionnaire (EDE-Q).***

The psychopathology of eating disorders (EDs) was assessed using the EDE-Q (Fairburn & Beglin, 1994). The German version contains 22 items across the four subscales *Restraint, Eating Concern, Weight Concern* and *Shape Concern* (Hilbert & Tuschen-Caffier, 2016)*.* Items are rated on a 6-point Likert scale based on the frequency of certain behaviors in the past 28 days (*0* = *no day* to *6 = every day*) or the severity of the behaviors (*0* = *not at all* to *6 = very much*), with higher scores indicating higher ED pathology. The internal consistency in the present study lay at Cronbach’s α = .98.

***Rosenberg Self-Esteem Scale (RSES).***

General self-esteem was assessed using the RSES (Rosenberg, 1965). The German version encompasses 10 items rated on a 4-point Likert scale (*0* = *strongly disagree* to *3 = strongly agree*), with higher scores indicating higher self-esteem (Ferring & Filipp, 1996). The internal consistency in this study lay at Cronbach’s α = .96.

***Upward Physical Appearance Comparison Scale (UPACS).***

Upward physical appearance comparisons were assessed using the UPACS (O'Brien et al., 2009). The German version of the scale consists of ten items rated on a 5-point Likert scale (*1 = strongly disagree* to *5 = strongly agree*), with higher scores indicating a higher tendency for upward appearance comparisons (Schönhals et al., 2024). The internal consistency in this study lay at Cronbach’s α = .90.

**Additional data analyses**

The additional data analyses were performed using IBM SPSS (version 28.0.1.1). To assess differences between women without EDs and with self-reported anorexia and bulimia nervosa (AN/BN) on baseline measures, *t*-tests were calculated for age, body mass index (BMI), and EDE-Q, RSES, and UPACS scores. Furthermore, a *t*-test was conducted to compare the sum of reported social media events during the seven-day study between women with and without EDs. When Levene’s test for homoscedasticity was significant (*p* < .10), Welch corrections are reported. These analyses were computed for the participants who were included in the final study sample.

Additionally, to test for differences between participants who completed the participant training and started the EMA and those who completed the baseline measures but did not start the EMA, *t*-tests were calculated for age, BMI, and EDE-Q, RSES, and UPACS scores. Furthermore, a *t*-test was conducted to compare the self-reported average daily time spent on social media between these two groups. When Levene’s test for homoscedasticity was significant (*p* < .10), Welch corrections are reported. The analyses were computed separately for women without EDs and for women with self-reported AN/BN who completed the baseline questionnaire (*n* = 244).

**Additional results regarding baseline measures and frequency of social media events**

The additional results can be derived from Supplement Table 1. There was no significant difference in age between women with self-reported AN/BN and without EDs. Women with AN/BN had a significantly lower BMI (*p* <. 001), significantly higher scores on the EDE-Q (*p* < .001) and UPACS (*p* < .001), and significantly lower scores on the RSES (*p* < .001), indicating higher ED pathology, a higher tendency for upward physical appearance comparisons, and lower self-esteem compared to women without EDs. Furthermore, women with AN/BN reported significantly more social media events than did women without EDs during the seven-day study period (*p* = .047).

In total, 17.9 % of women without EDs and 44.9 % of women with self-reported AN/BN did not start the EMA. There were no significant differences in any baseline measure between women who began the EMA and those who did not, either among women without EDs or among women with self-reported AN/BN (all *p* > .10, see Tables 2 and 3).

**References**

Fairburn, C. G., & Beglin, S. J. (1994). Assessment of eating disorders: interview or self-report questionnaire? *International Journal of Eating Disorders*, *16*(4), 317–417.

Ferring, D., & Filipp, S.‑H. (1996). Messung des Selbstwertgefühls: Befunde zu Reliabilität, Validität und Stabilität der Rosenberg-Skala. *Diagnostica*, *42*(3), 284–292.

Hilbert, A., & Tuschen-Caffier, B. (2016). *Eating Disorder Examination-Questionnaire: Deutschsprachige Übersetzung* (2. Auflage). dgvt-Verlag.

O'Brien, K. S., Caputi, P., Minto, R., Peoples, G., Hooper, C., Kell, S., & Sawley, E. (2009). Upward and downward physical appearance comparisons: Development of scales and examination of predictive qualities. *Body Image*, *6*(3), 201–206. https://doi.org/10.1016/j.bodyim.2009.03.003

Rosenberg, M. (1965). *Society and the adolescent self-image*. Princeton, NJ: Princeton University Press.

Schönhals, K., Quittkat, H. L., Voges, M. M., Ladwig, G., Holtmann, F.‑J., & Vocks, S. (2024). Is my body better than yours? Validation of the German version of the Upward and Downward Physical Appearance Comparison Scales in individuals with and without eating disorders. *Frontiers in Psychology*, *15*, 1390063. https://doi.org/10.3389/fpsyg.2024.1390063

**Supplement A Table 1**

*T-tests for differences between women with and without EDs.*

| Variable | Women without EDs (*n* = 81) | | Women with AN/BN (*n* = 62) | | *t* | *df* | *p* | Cohen’s *d* |
| --- | --- | --- | --- | --- | --- | --- | --- | --- |
|  | *M* | *SD* | *M* | *SD* |  |  |  |  |
| Age | 22.72 | 4.60 | 23.27 | 4.56 | 0.72 | 141 | .472 | 0.12 |
| BMI | 22.32 | 3.31 | 18.60 | 3.91 | -6.17 | 141 | <.001 | -1.04 |
| EDE-Q | 1.36 | 1.08 | 4.30 | 1.13 | 15.80 | 141 | <.001 | 2.67 |
| UPACS^a^ | 3.45 | 0.81 | 4.04 | 0.56 | 5.14 | 139.62 | <.001 | 0.83 |
| RSES | 2.20 | 0.67 | 0.86 | 0.54 | -12.88 | 141 | <.001 | -2.17 |
| Social media events | 14.58 | 9.61 | 17.66 | 8.38 | 2.01 | 141 | .047 | 0.34 |

*Note.* Age in years. BMI = Body mass index in kg/m^2^. EDE-Q = Eating Disorder Examination Questionnaire. UPACS = Upward Physical Appearance Comparison Scale. RSES = Rosenberg Self-Esteem Scale. Social media events = reported social media events during the seven-day study period. AN/BN = anorexia or bulimia nervosa. ED = eating disorder.

^a^Welch correction due to violation of homoscedasticity.

**Supplement A Table 2**

*T-tests for differences between women without eating disorders who started the EMA and who did not.*

| Variable | Started EMA  (*n* = 87) | | Did not start EMA (*n* = 19) | | *t* | *df* | *p* | Cohen’s *d* |
| --- | --- | --- | --- | --- | --- | --- | --- | --- |
|  | *M* | *SD* | *M* | *SD* |  |  |  |  |
| Age | 22.74 | 4.53 | 23.26 | 3.33 | 0.48 | 104 | .63 | 0.12 |
| BMI^a^ | 22.29 | 3.23 | 23.24 | 5.54 | 0.72 | 20.76 | .48 | 0.25 |
| EDE-Q | 1.34 | 1.06 | 1.72 | 1.36 | 1.33 | 104 | .19 | 0.34 |
| UPACS | 3.47 | 0.80 | 3.38 | 0.95 | -0.41 | 104 | .68 | -0.11 |
| RSES | 2.20 | 0.65 | 2.03 | 0.76 | -1.02 | 104 | .31 | -0.26 |
| Time spent on social media | 96.32 | 61.74 | 106.42 | 69.59 | 0.63 | 104 | .53 | 0.16 |

*Note.* Age in years. BMI = Body mass index in kg/m^2^. EDE-Q = Eating Disorder Examination Questionnaire. UPACS = Upward Physical Appearance Comparison Scale. RSES = Rosenberg Self-Esteem Scale. Time spent on social media = self-reported average daily time on social media in minutes.

^a^Welch correction due to violation of homoscedasticity.

**Supplement A Table 3**

*T-tests for differences between women with self-reported anorexia or bulimia nervosa who started the EMA and who did not.*

| Variable | Started EMA  (*n* = 76) | | Did not start EMA (*n* = 62) | | *t* | *df* | *p* | Cohen’s *d* |
| --- | --- | --- | --- | --- | --- | --- | --- | --- |
|  | *M* | *SD* | *M* | *SD* |  |  |  |  |
| Age | 23.20 | 4.34 | 23.79 | 4.84 | 0.76 | 136 | .45 | 0.13 |
| BMI | 18.37 | 3.86 | 19.48 | 4.24 | 1.61 | 136 | .11 | 0.28 |
| EDE-Q | 4.29 | 1.13 | 4.42 | 1.01 | 0.71 | 136 | .48 | 0.12 |
| UPACS | 4.01 | 0.57 | 4.10 | 0.65 | 0.87 | 136 | .38 | 0.15 |
| RSES | 0.88 | 0.55 | 0.97 | 0.52 | 0.99 | 136 | .33 | 0.17 |
| Time spent on social media | 112.46 | 91.43 | 102.56 | 64.21 | -0.72 | 136 | .47 | -0.12 |

*Note.* Age in years. BMI = Body mass index in kg/m^2^. EDE-Q = Eating Disorder Examination Questionnaire. UPACS = Upward Physical Appearance Comparison Scale. RSES = Rosenberg Self-Esteem Scale. Time spent on social media = self-reported average daily time spent on social media in minutes.
