# Supplementary material for: Fitspiration, Thinspiration, Body Positivity, and Body Neutrality Contents on Image‐Based Social Media: Associations With Body Image, Mood, Self‐Esteem, and Disordered Eating Behavior in Women With and Without Self‐Reported Eating Disorders—An Ecological Momentary Assessment Study
Source: Int J Eat Disord. 2026 Jan 8;59(4):790–802. doi: 10.1002/eat.70027 (PMC13058400; doi:10.1002/eat.70027)
Supplement: Supplementary file 2 — Data S2: Supplement B. [file EAT-59-790-s001.docx]

**Supplement B**

**Supplement B Figure 1**


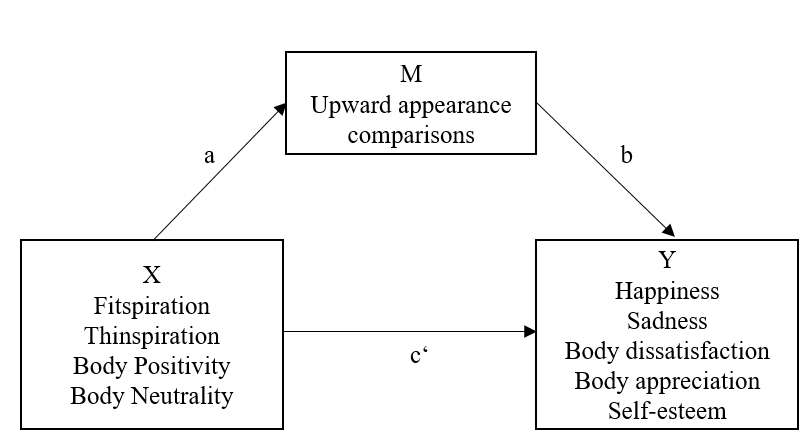
*Pathways in the mediation models*

*Note.* This figure demonstrates the pathways in the mediation models of Supplement B Table 1 to Table 5. X = independent variable, M = mediator, Y = dependent variable. Within-person effects are reported.

**Supplement B Table 1**

*Full mediation models of upward appearance comparisons during social media exposure for the effects of fitspiration, thinspiration, body positivity, and body neutrality on happiness.*

| Antecedent | Upward appearance comparisons (M) | | | | Happiness (Y) | | | | |
| --- | --- | --- | --- | --- | --- | --- | --- | --- | --- |
|  | Path | Estimate | Est. Error | *p* | Path | Estimate | Est. Error | *p* | *d* [95% CI] |
| Fitspiration (X) | a | 0.57 | 0.04 | <.001 | c‘ | -1.60 | 0.82 | .053 |  |
| Upward appearance comparisons (M) |  |  |  |  | b | -2.26 | 0.37 | <.001 |  |
| Indirect effect |  |  |  |  | a*b | -1.28 | 0.23 | <.001 | -0.07 [-0.09, -0.04] |
| Total effect |  |  |  |  | a*b+c‘ | -2.88 | 0.80 | <.001 |  |
| Thinspiration (X) | a | 0.61 | 0.06 | <.001 | c‘ | -2.39 | 1.04 | .020 |  |
| Upward appearance comparisons (M) |  |  |  |  | b | -2.26 | 0.37 | <.001 |  |
| Indirect effect |  |  |  |  | a*b | -1.38 | 0.26 | <.001 | -0.07 [-0.10, -0.05] |
| Total effect |  |  |  |  | a*b+c‘ | -3.76 | 1.02 | <.001 |  |
| Body positivity (X) | a | 0.09 | 0.05 | .065 | c‘ | 0.41 | 0.90 | .646 |  |
| Upward appearance comparisons (M) |  |  |  |  | b | -2.26 | 0.37 | <.001 |  |
| Indirect effect |  |  |  |  | a*b | -0.21 | 0.12 | .065 | -0.01 [-0.02, 0.00] |
| Total effect |  |  |  |  | a*b+c‘ | 0.20 | 0.91 | .829 |  |
| Body neutrality (X) | a | 0.10 | 0.05 | .072 | c‘ | 1.55 | 0.95 | .104 |  |
| Upward appearance comparisons (M) |  |  |  |  | b | -2.26 | 0.37 | <.001 |  |
| Indirect effect |  |  |  |  | a*b | -0.22 | 0.13 | .072 | -0.01 [-0.03, 0.00] |
| Total effect |  |  |  |  | a*b+c‘ | 1.33 | 0.95 | .162 |  |

*Note.* The pathways are depicted in Supplement B Figure 1. X = independent variable, M = mediator, Y = dependent variable, CI = confidence interval.

**Supplement B Table 2**

*Full mediation models of upward appearance comparisons during social media exposure for the effects of fitspiration, thinspiration, body positivity, and body neutrality on sadness.*

| Antecedent | Upward appearance comparisons (M) | | | | Sadness (Y) | | | | |
| --- | --- | --- | --- | --- | --- | --- | --- | --- | --- |
|  | Path | Estimate | Est. Error | *p* | Path | Estimate | Est. Error | *p* | *d* [95% CI] |
| Fitspiration (X) | a | 0.57 | 0.05 | <.001 | c‘ | -0.30 | 0.83 | .728 |  |
| Upward appearance comparisons (M) |  |  |  |  | b | 2.49 | 0.37 | <.001 |  |
| Indirect effect |  |  |  |  | a*b | 1.41 | 0.24 | <.001 | 0.07 [0.05, 0.10] |
| Total effect |  |  |  |  | a*b+c‘ | 1.12 | 0.82 | .172 |  |
| Thinspiration (X) | a | 0.61 | 0.06 | <.001 | c‘ | 4.47 | 1.04 | <.001 |  |
| Upward appearance comparisons (M) |  |  |  |  | b | 2.49 | 0.37 | <.001 |  |
| Indirect effect |  |  |  |  | a*b | 1.52 | 0.27 | <.001 | 0.08 [0.05, 0.11] |
| Total effect |  |  |  |  | a*b+c‘ | 5.99 | 1.03 | <.001 |  |
| Body positivity (X) | a | 0.09 | 0.05 | .069 | c‘ | -0.73 | 0.91 | .421 |  |
| Upward appearance comparisons (M) |  |  |  |  | b | 2.49 | 0.37 | <.001 |  |
| Indirect effect |  |  |  |  | a*b | 0.23 | 0.13 | .069 | 0.01 [0.00, 0.03] |
| Total effect |  |  |  |  | a*b+c‘ | -0.50 | 0.92 | .590 |  |
| Body neutrality (X) | a | 0.10 | 0.05 | .064 | c‘ | -0.98 | 0.97 | .310 |  |
| Upward appearance comparisons (M) |  |  |  |  | b | 2.49 | 0.37 | <.001 |  |
| Indirect effect |  |  |  |  | a*b | 0.24 | 0.14 | .064 | 0.01 [0.00, 0.03] |
| Total effect |  |  |  |  | a*b+c‘ | -0.73 | 0.98 | .458 |  |

*Note.* The pathways are depicted in Supplement B Figure 1. X = independent variable, M = mediator, Y = dependent variable, CI = confidence interval.

**Supplement B Table 3**

*Full mediation models of upward appearance comparisons during social media exposure for the effects of fitspiration, thinspiration, body positivity, and body neutrality on body dissatisfaction.*

| Antecedent | Upward appearance comparisons (M) | | | | Body dissatisfaction (Y) | | | | |
| --- | --- | --- | --- | --- | --- | --- | --- | --- | --- |
|  | Path | Estimate | Est. Error | *p* | Path | Estimate | Est. Error | *p* | *d* [95% CI] |
| Fitspiration (X) | a | 0.57 | 0.05 | <.001 | c‘ | 1.96 | 0.76 | .009 |  |
| Upward appearance comparisons (M) |  |  |  |  | b | 2.81 | 0.34 | <.001 |  |
| Indirect effect |  |  |  |  | a*b | 1.60 | 0.24 | <.001 | 0.08 [0.06, 0.10] |
| Total effect |  |  |  |  | a*b+c‘ | 3.55 | 0.74 | <.001 |  |
| Thinspiration (X) | a | 0.61 | 0.06 | <.001 | c‘ | 4.49 | 0.94 | <.001 |  |
| Upward appearance comparisons (M) |  |  |  |  | b | 2.81 | 0.34 | <.001 |  |
| Indirect effect |  |  |  |  | a*b | 1.72 | 0.27 | <.001 | 0.09 [0.06, 0.12] |
| Total effect |  |  |  |  | a*b+c‘ | 6.21 | 0.93 | <.001 |  |
| Body positivity (X) | a | 0.09 | 0.05 | .060 | c‘ | -1.47 | 0.86 | .084 |  |
| Upward appearance comparisons (M) |  |  |  |  | b | 2.81 | 0.34 | <.001 |  |
| Indirect effect |  |  |  |  | a*b | 0.26 | 0.15 | .060 | 0.01 [0.00, 0.03] |
| Total effect |  |  |  |  | a*b+c‘ | -1.21 | 0.87 | .163 |  |
| Body neutrality (X) | a | 0.10 | 0.05 | .072 | c‘ | -1.62 | 0.87 | .061 |  |
| Upward appearance comparisons (M) |  |  |  |  | b | 2.81 | 0.34 | <.001 |  |
| Indirect effect |  |  |  |  | a*b | 0.28 | 0.16 | .072 | 0.01 [0.00, 0.03] |
| Total effect |  |  |  |  | a*b+c‘ | -1.34 | 0.88 | .125 |  |

*Note.* The pathways are depicted in Supplement B Figure 1. X = independent variable, M = mediator, Y = dependent variable, CI = confidence interval.

**Supplement B Table 4**

*Full mediation models of upward appearance comparisons during social media exposure for the effects of fitspiration, thinspiration, body positivity, and body neutrality on body appreciation.*

| Antecedent | Upward appearance comparisons (M) | | | | Body appreciation (Y) | | | | |
| --- | --- | --- | --- | --- | --- | --- | --- | --- | --- |
|  | Path | Estimate | Est. Error | *p* | Path | Estimate | Est. Error | *p* | *d* [95% CI] |
| Fitspiration (X) | a | 0.57 | 0.05 | <.001 | c‘ | -2.49 | 0.68 | <.001 |  |
| Upward appearance comparisons (M) |  |  |  |  | b | -2.22 | 0.31 | <.001 |  |
| Indirect effect |  |  |  |  | a*b | -1.26 | 0.20 | <.001 | -0.06 [-0.09, -0.04] |
| Total effect |  |  |  |  | a*b+c‘ | -3.75 | 0.67 | <.001 |  |
| Thinspiration (X) | a | 0.61 | 0.06 | <.001 | c‘ | -2.40 | 0.86 | .006 |  |
| Upward appearance comparisons (M) |  |  |  |  | b | -2.22 | 0.31 | <.001 |  |
| Indirect effect |  |  |  |  | a*b | -1.36 | 0.23 | <.001 | -0.07 [-0.09, -0.05] |
| Total effect |  |  |  |  | a*b+c‘ | -3.76 | 0.86 | <.001 |  |
| Body positivity (X) | a | 0.09 | 0.05 | .075 | c‘ | 0.88 | 0.76 | .240 |  |
| Upward appearance comparisons (M) |  |  |  |  | b | -2.22 | 0.31 | <.001 |  |
| Indirect effect |  |  |  |  | a*b | -0.21 | 0.12 | .075 | -0.01 [-0.02, 0.00] |
| Total effect |  |  |  |  | a*b+c‘ | 0.67 | 0.77 | .380 |  |
| Body neutrality (X) | a | 0.10 | 0.05 | .068 | c‘ | 1.93 | 0.79 | .012 |  |
| Upward appearance comparisons (M) |  |  |  |  | b | -2.22 | 0.31 | <.001 |  |
| Indirect effect |  |  |  |  | a*b | -0.22 | 0.13 | .068 | -0.01 [-0.02, 0.00] |
| Total effect |  |  |  |  | a*b+c‘ | 1.71 | 0.80 | .032 |  |

*Note.* The pathways are depicted in Supplement B Figure 1. X = independent variable, M = mediator, Y = dependent variable, CI = confidence interval.

**Supplement B Table 5**

*Full mediation models of upward appearance comparisons during social media exposure for the effects of fitspiration, thinspiration, body positivity, and body neutrality on self-esteem.*

| Antecedent | Upward appearance comparisons (M) | | | | Self-esteem (Y) | | | | |
| --- | --- | --- | --- | --- | --- | --- | --- | --- | --- |
|  | Path | Estimate | Est. Error | *p* | Path | Estimate | Est. Error | *p* | *d* [95% CI] |
| Fitspiration (X) | a | 0.57 | 0.04 | <.001 | c‘ | -0.02 | 0.03 | .526 |  |
| Upward appearance comparisons (M) |  |  |  |  | b | -0.11 | 0.01 | <.001 |  |
| Indirect effect |  |  |  |  | a*b | -0.06 | 0.01 | <.001 | -0.07 [-0.09, -0.05] |
| Total effect |  |  |  |  | a*b+c‘ | -0.08 | 0.03 | .006 |  |
| Thinspiration (X) | a | 0.61 | 0.06 | <.001 | c‘ | -0.08 | 0.04 | .050 |  |
| Upward appearance comparisons (M) |  |  |  |  | b | -0.11 | 0.01 | <.001 |  |
| Indirect effect |  |  |  |  | a*b | -0.07 | 0.01 | <.001 | -0.07 [-0.10, -0.05] |
| Total effect |  |  |  |  | a*b+c‘ | -0.14 | 0.04 | <.001 |  |
| Body positivity (X) | a | 0.09 | 0.05 | .056 | c‘ | 0.07 | 0.03 | .041 |  |
| Upward appearance comparisons (M) |  |  |  |  | b | -0.11 | 0.01 | <.001 |  |
| Indirect effect |  |  |  |  | a*b | -0.01 | 0.01 | .056 | -0.01 [-0.02, 0.00] |
| Total effect |  |  |  |  | a*b+c‘ | 0.06 | 0.03 | .087 |  |
| Body neutrality (X) | a | 0.10 | 0.05 | .068 | c‘ | 0.00 | 0.04 | .992 |  |
| Upward appearance comparisons (M) |  |  |  |  | b | -0.11 | 0.01 | <.001 |  |
| Indirect effect |  |  |  |  | a*b | -0.01 | 0.01 | .068 | -0.01 [-0.03, 0.00] |
| Total effect |  |  |  |  | a*b+c‘ | -0.01 | 0.04 | .771 |  |

*Note.* The pathways are depicted in Supplement B Figure 1. X = independent variable, M = mediator, Y = dependent variable, CI = confidence interval.
